# Identification of gene targets against dormant phase *Mycobacterium tuberculosis *infections

**DOI:** 10.1186/1471-2334-7-84

**Published:** 2007-07-26

**Authors:** Dennis J Murphy, James R Brown

**Affiliations:** 1 Informatics, Molecular Discovery Research, GlaxoSmithKline, 1250 South Collegeville Road, UP1345, PO Box 5089, Collegeville, PA 19426-0989, USA; 2 Department of Biochemistry, UW2523, Cardiovascular and Urogenital CEDD, GlaxoSmithKline, 709 Swedeland Road, Box 1539, King of Prussia, PA 19406, USA

## Abstract

**Background:**

*Mycobacterium tuberculosis*, the causative agent of tuberculosis (TB), infects approximately 2 billion people worldwide and is the leading cause of mortality due to infectious disease. Current TB therapy involves a regimen of four antibiotics taken over a six month period. Patient compliance, cost of drugs and increasing incidence of drug resistant *M. tuberculosis *strains have added urgency to the development of novel TB therapies. Eradication of TB is affected by the ability of the bacterium to survive up to decades in a dormant state primarily in hypoxic granulomas in the lung and to cause recurrent infections.

**Methods:**

The availability of *M. tuberculosis *genome-wide DNA microarrays has lead to the publication of several gene expression studies under simulated dormancy conditions. However, no single model best replicates the conditions of human pathogenicity. In order to identify novel TB drug targets, we performed a meta-analysis of multiple published datasets from gene expression DNA microarray experiments that modeled infection leading to and including the dormant state, along with data from genome-wide insertional mutagenesis that examined gene essentiality.

**Results:**

Based on the analysis of these data sets following normalization, several genome wide trends were identified and used to guide the selection of targets for therapeutic development. The trends included the significant up-regulation of genes controlled by *devR*, down-regulation of protein and ATP synthesis, and the adaptation of two-carbon metabolism to the hypoxic and nutrient limited environment of the granuloma. Promising targets for drug discovery were several regulatory elements (*devR/devS*, *relA*, *mprAB*), enzymes involved in redox balance and respiration, sulfur transport and fixation, pantothenate, isoprene, and NAD biosynthesis. The advantages and liabilities of each target are discussed in the context of enzymology, bacterial pathways, target tractability, and drug development.

**Conclusion:**

Based on our bioinformatics analysis and additional discussion of in-depth biological rationale, several novel anti-TB targets have been proposed as potential opportunities to improve present therapeutic treatments for this disease.

## Background

*Mycobacterium tuberculosis*, the causative agent of tuberculosis (TB), kills more than 2 million people per year and has infected an estimated 2 billion people worldwide. It is the leading cause of mortality due to infectious disease [1]. The host immune response to aerosol infection is to quarantine *M. tuberculosis *in a structure called a granuloma which halts replication of the bacillus and suppresses the immediate threat of active infection [2]. However, granuloma associated *M. tuberculosis *bacterium can switch to a dormant or non-replicative state and successfully evade any immune response for decades post-initial infection [3]. As the host immune system falters, *M. tuberculosis *returns to replication mode, which leads to the recurrence of active infection. Thus current TB therapy to fight off active disease requires a strictly monitored treatment period or DOTS (directly observed treatment, short course) lasting up to six months and involving four different drugs: isoniazid, rifampicin, pyrazinamide, and ethambutol [4-6]. Patient compliance with this prolonged therapeutic regime is an important concern. Moreover, prolonged exposure to drugs has likely been an important factor behind increasing reports of anti-biotic resistant bacterium [7]. The lack of well-defined targets specific to dormancy phase *M. tuberculosis *has been a major obstacle in the development of effective short-course therapies.

A number of studies have attempted to develop *in vitro *and *in vivo *models of non-replicating, dormant phase *M. tuberculosis*. These can be grouped into four main types: hypoxia, starvation, macrophages, and murine infection. Each system mimics some of, but not the entire, clinical situation. The Wayne model (slowly stirred, sealed cultures with a defined air-space to medium ratio) [8] captures the presumed hypoxic nature of the granuloma, but lacks the effect of the immune response, macrophage phagocytocis, and eventual release to the extracellular milieu. The starvation models are not hypoxic and may not capture the unbalanced diet in the granuloma. The macrophage phagocytosis experiments show the early adaptation to the host immune response, but do not address the long term metabolic changes. While the murine model replicates many facets of the human immune response [9], mice do not show well-formed granulomas and lack the caseous, necrotic centers characteristic of human infection [10]. None of these models fully capture the heterogeneity of the granuloma, with a gradient of active and inactive/dead immune cells, oxygenation, and nutrients.

DNA microarrays have been used to determine the complete transcriptional response of *M. tuberculosis *cultured in each of these experimental models. In addition to the dormancy models, DNA microarrays have also been employed to do genome-scale knockout experiments using saturating transposon insertion mutagenesis. Mutants have been profiled for the ability to grow *in vitro*, in mouse macrophages, and *in vivo *mouse models [11-14]. In these experiments genes containing (presumably inactivating) insertions are selected for the ability to grow. A disrupted gene that inhibits growth yields a decreased signal on the microarray compared to the genomic control. In the absence of suitable gene inactivation studies for the dormant phase, the phenotypic effects of gene knock-outs on growth phase *M. tuberculosis *seems to be the best indicator of gene essentiality from a drug target perspective.

Here we present a novel and comprehensive meta-analysis of the *M. tuberculosis *gene expression and gene disruption microarray data sets. In the absence of a perfect experimental model we chose a consensus approach and combined the different analyses together into a single database. Recently, Hasan *et al*. of the pharmaceutical company, Novartis, described an in-house software tool for *in silico *prioritizing of genomic drug targets in pathogens and illustrated its use on *M. tuberculosis *[15]. Using this tool, they provided three lists based on different prioritization criteria for: 1) genes associated with critical metabolic reactions (chokepoints) unique to *M. tuberculosis*; 2) genes highly specific to the Actinobacter (the taxonomic class of bacteria inclusive of *M. tuberculosis*) and absent from other gut flora and 3) genes potentially important in maintaining persistence. The latter criterion is the most critical clinical need for TB treatment, and both Hasan's *et al*. study and ours use several similar published gene expression and essential data-sets to approach the identification of *M. tuberculosis *persistence targets. However, GlaxoSmithKline experiences in developing novel antibacterials from a genomics-driven target-based approach have shown the importance of multiple analysis perspectives and the need to substantiate initial bioinformatics target identifications with additional biological rationale [16]. Therefore, while encouraged that several potential targets presented here are also substantiated by earlier computational studies using different approaches, we feel it is important to further extend and rationalize *in silico *target validation with current knowledge on the biology, biochemistry and disease pathology of *M. tuberculosis*. Thus, we have tried to provide such additional rationale when evaluating those targets from our prioritized list that are the most promising candidates for concerted drug development programs.

## Results

**Figure 1:**
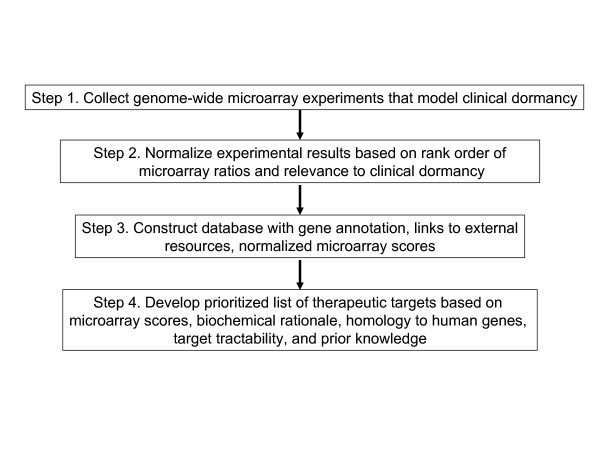
Flowchart of the process used to generate the prioritized list of tractable therapeutic targets.

Figure 1 shows a flowchart of our approach to utilize the microarray data sets to identify putative gene targets in non-replicative *M. tuberculosis*. Table 1 shows the data sets collected in the first step. Since the experimental conditions in the dormancy models were quite varied (e.g. 24 h of starvation in culture media to 4 weeks in whole mice), the expression results for each gene were normalized (Fig. 1, Step 2). A zero to five scoring system was developed based upon two criteria. The first criterion was the overall relevance of the experimental conditions to persistance in the granuloma. The mouse macrophage and whole animals studies model the immediate response of *M. tuberculosis *to immune attack and long term survival in the host. The granuloma itself is characterized by avascularization and necrosis which have been modeled by the hypoxic and starvation conditions. The maximum score for a particular experimental dataset was adjusted based on potential relevance to the clinical occurrence of dormancy phase *M. tuberculosis *infections. For studies with multiple time series sampling, increasing weight was given to later time-point samples. The second criteria involved the rank order of gene expression in a particular study which allowed for cross-study comparsions (See Table 1 and Methods for details on the scoring scheme). Down-regulated expression was scored the same as up-regulated expression except that negative values were used to easily separate the two sets of results. (Some genes show significant scores in both the up-regulated and down-regulated data sets. This is not surprising considering the variation among studies in experimental situation and the number of time-points.) The knockout experiments were similarly scored by rank order of effect on growth, except that genes having no effect were scored as zero.

**Table 1:** Sources, experimental models, and scoring criteria for *Mycobacterium tuberculosis *DNA microarray gene expression and genome-wide gene knock-out (growth phase essentiality) data used in this study.

**Reference**	**Experimental model**	**Timepoint: Maximum score^a^**
Betts *et al*., 2002 [116]	Starvation under controlled O_2_	96 h: 3 24 h: 2 4 h: 1
Hampshire *et al*., 2004 [30]	Nutrient depletion under controlled O_2_	62 and 75 d: 5 49 d: 4 18 d: 2
Muttucumaru *et al*., 2004 [115]	Wayne model of hypoxia [8]	14 d (NRP-2): 4 7 d (NRP-1): 2
Voskuil *et al*. 2004 [31]	Wayne model of hypoxia [8]	30 and 80 d: 5 14 and 20 d: 4 10 and 12 d: 3 6 and 8 d: 2
Schnappinger *et al*. 2003 [29]	Infection of mouse macrophages, +/- γ-INF	24 and 48 h: 5
Karakousis *et al*., 2004 [117]	Hollow fiber subcutaneous implant in mice	10 d: 3
Talaat *et al*., 2004 [118]	Infection of mice. MTB harvested from lung^b^	28 d: 3
Sassetti *et al*. 2003a [13]	TraSH mutated libraries grown on solid media	14 d:5
Rengarajan *et al*. 2005 [12]	Infection of mouse macrophages, +/- γ-INF with TraSH mutated libraries of *M. tuberculosis*	7 d: 5
Sassetti *et al*. 2003b [14]	C57BL/6J mice infected with TraSH mutated libraries of *M. tuberculosis*	7, 14, 28 and 56 d: 5

Footnotes

^a^Maximum score based on relevance as a dormancy model.

^b^Ratio of *M. tuberculosis *from Balb/c lung to MTB in aerated culture for 28 d.

In Step 3 scores for each gene in each of the experimental conditions were collected into a Microsoft Access database. Reference fields were added to facilitate prioritization, such as the Refseq ID, Genbank function, Genbank note, Tuberculist classification, and KEGG and Sanger Center links. These data are available in Additional file 1.

**Figure 2:**
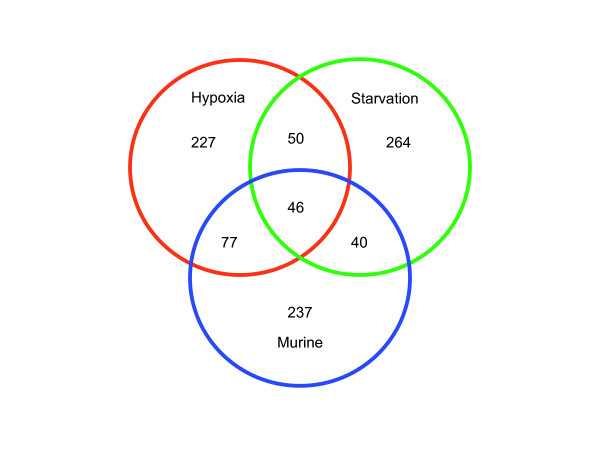
Overlap of the top 400 highest-scoring genes (~10% of the genome) from each of the three types of experimental models of dormancy. Murine refers to *M. tuberculosis *cells isolated from mouse macrophages, subcutaneous hollow fiber, and lung.

There are two important characteristics of this meta-analysis: i) in order for a gene to score well it must be in the top quarter of highly induced genes across several experimental models of dormancy and ii) the expression levels for the highest expressing genes are attenuated. The latter item has the effect of avoiding the situation where a very large fold increase in one experiment dwarfs all other results. The first point is illustrated by the intersection of the top 400 genes (~10% of the genome) from the hypoxia, starvation, and *in vivo *murine models, shown in Fig. 2. The great majority of the high scoring genes come from the subset where two or three of the groups intersect. By combining the data from different models, a consensus view can reached about the particular genes and pathways most critical for survival in the dormant state.

### Multi-gene trends

**Figure 3:**
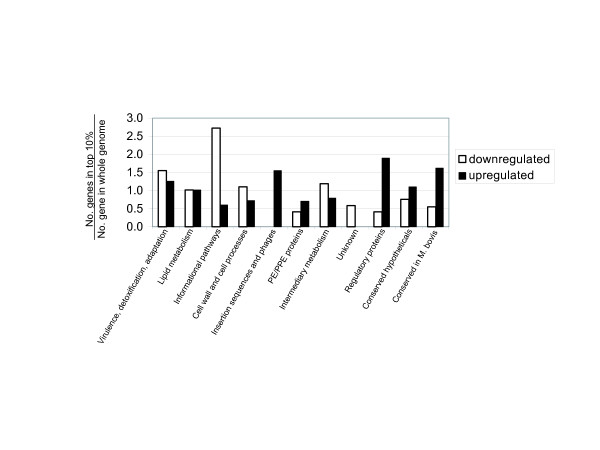
Ratio of the number of genes in the highest-scoring fraction (top 10%) to the number in the entire genome, for each classification described in Cole, *et al*. [17]. Ratios greater than 1 indicate that the genes are over-represented in the highest-scoring fraction relative to their representation in the whole genome.

Figure 3 shows a comparison of the functional classes [17] of the up-regulated and down-regulated genes to the whole genome. The proportion of genes in the top 10% of up or down-regulated genes was divided by the proportion of that functional class in the entire genome and the ratio is plotted. The following sections highlight differential changes in particular functional classes shown in Fig. 3 as well as other multi-gene expression results that impacted the selection of therapeutic targets.

### *devR *regulon

**Figure 4:**
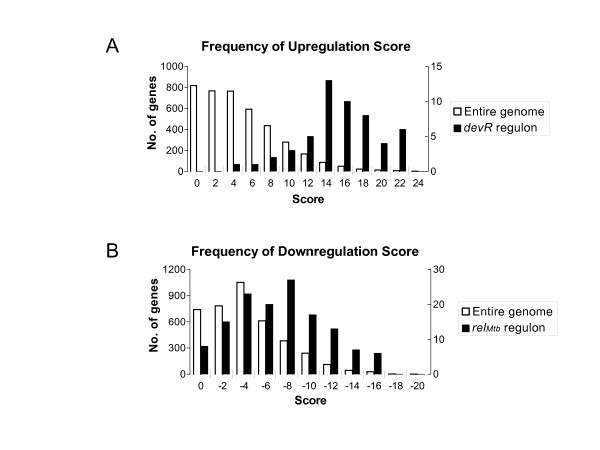
Histogram of the distribution of upregulated and downregulated gene scores for the entire genome overlaid with the distribution from the *devR *regulon (A) and the *relA *regulon (B).

There is significant experimental evidence that the transcriptional regulator *devR *(also called *dosR*) is a key factor in the metabolic shift-down to non-replicative persistence (see Discussion). Based on the 20 nt consensus sequence of *devR *and microarray expression results, two studies have identified 53 genes that appear to be induced in response to *devR *activation [18,19]. The analysis here shows 81% of the 53 genes in the *devR *regulon are in the top 5% of highest scoring genes, confirming the importance of this regulatory element in dormancy under a variety of experimental conditions (see Additional file 2). Only up-regulated genes were extracted from the data sets [18,19] so it is expected that these 53 genes show higher than average up-regulation scores (see Kendall [20] and Ohno [21] for analyses that included genes down-regulated by *devR*). Fig. 4A shows a comparison of the expression pattern for the entire genome and the *devR *regulon.

While the 53 genes regulated by *devR *appear to play an important role in dormancy, nearly 60% of the genes do not have an annotated function. In order to gain some insight into the genes regulated by this system, we searched against INTERPRO (version 12.0) and the COGS database [22,23]. While some of the assignments are speculative, (i.e. based on partial overlap with a domain of known function [24]) several useful clues emerge. Eleven genes are involved in carbohydrate and fatty acid metabolism, and eight genes function in electron transfer. We suggest these genes reflect the biased nutrient pool and lack of oxygen, requiring altered pathways for biosynthesis and to generate oxidizable metabolites and utilization of other terminal electron acceptors. The occurrence of four transporters also indicates a limited nutrient pool. Among the other genes of the *devR *regulon, only two genes alter translation compared with possibly seven transcriptional regulators. This makes the *devR *regulon even more significant when one considers the cascading signal that is produced. These and the other regulators (36 of the 190 total are in the top 10%) show that *M. tuberculosis *has to make global changes to achieve a dormant state. There are several genes involved in nucleotide biosynthesis despite the fact that the genome has been replicated prior to entry into the non-replicative state [25]. This can be rationalized by the need to make repairs to maintain the integrity of the genome over decades [26]. The signal from the expression experiments for the universal stress proteins (USPs) is very strong: six of the eight USPs are part of the *devR *regulon, and five of those are in the top 5% of up-regulated genes.

Only six of the 53 genes have an effect on growth in the gene disruption experiments (TraSH), supporting the idea that their main role is in shifting to and maintaining the non-replicative state. *DevS*, a regulator of *devR *(see below) inhibits growth *in vitro *[13] and significantly in the mouse macrophage model [14], consistent with the response to nitric oxide [18], Disruption of one of the USPs inhibits *in vitro *growth [13]; the others had no effect. The gene with the highest growth inhibition score (9.4) was Rv2004c. This gene has a partial overlap and low similarity to gluconate kinase, but is highly similar to COG2187, a conserved bacterial domain of unknown function.

### C_2 _metabolism

Fig. 3 shows modest changes in the proportion of genes involved in intermediary metabolism and respiration. We interpret this to mean *M. tuberculosis *has kept a portion of its metabolic repertoire intact to adapt to hypoxia and a biased nutrient supply. Up-regulation of genes in C_2 _metabolism, especially enzymes that maintain redox balance, utilize alternate terminal electron acceptors, or handle the increased two-carbon flux point to these proteins as particularly important in the metabolic alterations made for survival in the hypoxic granuloma.

### Downregulation of ATP synthesis

Consistent with previous work and analysis [9,27] the data here shows a strong down-regulation of the F_1_-F_O _ATP synthase (see Additional file 3), a likely consequence of hypoxia and the utilization of other terminal electron acceptors. It thus seems probable that ATP is a scarce resource in the non-replicating cell.

### Down-regulation of ribosomal proteins

Hu *et al*. [28] showed a 98% decrease in protein synthesis using ^35^S-met pulse labeling experiments with microaerophilic cultures (50 days in unstirred, screwcap vials) similar to the Wayne model [8]. The transcriptional experiments collected here are consistent with this result. Over half of the 55 30S and 50S ribosomal-protein genes encoded by the *M. tuberculosis *genome are down-regulated. Fig. 3 also illustrates that information pathways (including transcription and translation) show significant down-regulation.

### Isoprene biosynthesis

**Figure 5:**
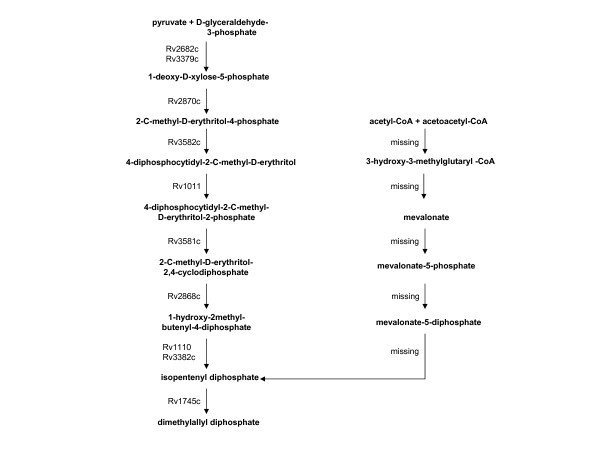
The two biosynthetic pathways for isopentenyl-pyrophosphate biosynthesis. The gene products (enzymes) from the *M. tuberculosis *genome presumed to catalyze each transformation are specified by the Rv numbers. Enzymes for which there is not a relevant orthologue in the *M. tuberculosis *genome are labeled as missing. [119] [76].

Fig. 5 shows the isopentenyl-pyrophosphate biosynthetic pathway in *M. tuberculosis*. Of all the metabolic pathways we examined, this pathway showed the most consistent up-regulation of genes across the entire pathway. One of the uses of isopentenyl-pyrophosphate is the biosynthesis of decaprenyl phosphate, which is needed for cell wall construction (see Discussion). Consistent with this role is the result that insertion into *dxs1*, *ispD*, *ispE *and *ispF *show inhibition in the *in vitro *growth experiments [13]. Significant synthesis of cell wall components is also needed to survive the multiple stresses inside the macrophage phagosome. Nearly all of genes in the pathway are up-regulated in the mouse macrophage model [29]. Also contributing to the high expression scores are the later time points in the long terms experiments [30,31]. This may reflect the continuing need to maintain membrane integrity during long term survival.

## Discussion

Our analysis suggests that during dormancy *M. tuberculosis *performs limited protein synthesis. The pathogen spends its available resources on maintaining cell wall, membrane potential and genome integrity as well as resisting host defenses.

The main objective of our analysis was to develop a prioritized list of genes that might be suitable targets for drugs effective against non-replicating bacilli (Fig 1, Step 4). The foremost assumption is that genes that are highly expressed in a number of experimental situations are important for persistence of non-replicating *M. tuberculosis*. This was not an absolute criterion but pathways or multimeric complexes in which only a single gene was up-regulated were judged to be less important or anomalous results and, therefore were considered to be lower priority targets. The knockout experiments address a fundamentally different question than do the dormancy experiments (e.g. replication versus non-replicative survival). The focus of this analysis is on the non-replicative state. Targets that show both up-regulation in the dormant state and inhibit growth are certainly more interesting, but it may well be the case that genes required for survival in the non-replicating state have no effect on growth. As an example, Rengarajan *et al*. found that genes expressed under hypoxic or low nutrient conditions simulating the granuloma environment, are not necessarily essential for intracellular growth in macrophages [12]. In bacteria, growth essential genes are often constitutively expressed while virulence and environmental response genes tend be highly regulated yet essential during those periods of high induction. Thus one would expect that different groups of genes play distinctive roles, and have transient importance for viability, in the non-replicative versus replicative phases of the bacterium's life cycle.

Here we discuss the relative tractability of proposed dormancy phase modulated genes and pathways as drug targets for the eradication of dormancy phase *M. tuberculosis*. The individual genes and scores discussed below are listed in Additional file 4. Since not every gene in a particular pathway scored highly, we included additional low scoring pathway proteins in order to provide context for targeting particular pathways.

### *devR *regulon and other regulatory systems

An effective immune response that halts disease progression typically results in formation of granulomas. It is in these granulomas that the non-replicative bacilli are believed to reside [32,33]. Based on the nature of the granuloma, it is very likely that the non-replicating bacilli are surviving in an hypoxic environment. Granulomas are necrotic, avascular lesions that are walled off from the immune system. They are made up of caseous (chalky) centers and contain dead macrophages, T-cells and the tubercular bacilli [34,35]. This environment also has ramifications for the nutrient supply.

The results here and elsewhere [18,19,36,37] clearly show that the *devR *regulon (also known as the *dosR *regulon) is a significant regulator of genes in response to the experimental stressors of hypoxia and nitric oxide. This, combined with the production of nitric oxide by host immune cells and the likely hypoxic nature of the granuloma, makes a strong, albeit circumstantial, case for this regulon to be a primary trigger in the metabolic shiftdown to achieve the non-replicative state.

Additional evidence comes from Malhotra *et al*. [38] where guinea pigs were infected with wild type H37Rv *M. tuberculosis *and a *devR*::*kan *mutant. After 47 days the lungs of the animals infected with the mutant strain showed small granulomas (<20%) and a normal lung architecture; the lungs of the animals infected with wild type showed partial to complete destruction with granulomas taking over 45–85% of the lung. Using the less virulent strain *Mycobacterium bovis *BCG, Boon and Dick showed that a *devR*::*kan *mutant grew at the same rate as wild type during the aerated phase of the Wayne model, but had a 1500 fold decrease in viability during the 5 week hypoxic phase [39]. Mice lacking iNOS are rapidly killed by *M. tuberculosis*, whereas mice with functional iNOS yield chronic infections. One interpretation is that nitric oxide activates *devS *and *devR*, resulting in metabolic downshifting and latent infection. Without the nitric oxide signal, the regulon is not upregulated and cell division continues until the host is killed [40,41].

#### *devS*/*devT *devR two component sensor

Two component response regulator systems are found in bacteria, fungi, and plants [42,43] and are already being exploited as new antibiotic targets [44,45]. These systems consist of a sensor kinase (component 1) which autophosphorylates histidine, then transphosphorylates a conserved aspartate of its cognate transcriptional regulator (component 2). *M. tuberculosis *has eleven complete systems and seven orphans belonging to sensor kinase and response regulator families [17]. It has been shown that recombinant sensor kinases *devS *and *devT *autophosphorylate to stable phosphoproteins, but rapidly transphosphorylate *devR *[46-48], confirming these as members of the two component family. Additionally several papers have identified *devS/devT devR *as a strong candidate for therapeutic intervention [15,47,49,6,52]. The results collected here are consistent with these analyses. It interesting to speculate that this approach may work not by killing *M. tuberculosis *directly, but by forcing them out of the non-replicative state, [2] leaving them susceptible to the usual anti-microbial agents. If the regulatory system having significant control over non-replicative metabolism were disrupted, this could lead to exit from the non-replicative state or a metabolic catastrophe. Ideally the cell would suffer unregulated expression with insufficient resources from which it could not recover. Paving the way for further work are recent papers which describe a validated high-throughput screening assay [49]. and x-ray crystal structures of the C-terminus of *devR*, with and without DNA bound. [50]. A fluorescence polarization assay has been described for binding of component 2 to its cognate DNA sequence [53].

A bifunctional inhibitor or a combination of compounds may be necessary to attack the sensor kinase since it took knockouts of both *devS *and *devT *to prevent phosphorylation of *devR *after 2 h of hypoxia [46]. Blocking *devR *binding to its cognate DNA-binding sites might require a less complex therapeutic strategy. The ATP site here and in the universal stress proteins and mprA, mprB regulators (below) offers the opportunity to take advantage of the growing expertise in development of kinase inhibitors [54].

### Universal stress proteins

Universal stress proteins (USPs) are a family of proteins that are produced by bacteria in response to a range of environmental stressors and cause a significant change in metabolism [55]. In our analysis, five of the eight *Usp *genes in the *M. tuberculosis *genome score in the upper 5% of expression results. The exact function of USPs in TB pathogenicity is not fully understood although studies in other species suggest they likely play a key cellular role. The function of UspA from *E. coli *has been characterized by Nyström *et al*. [56]. Increased expression of UspA to physiological levels caused a global change in protein expression and decreased cell growth on minimal media, and decreased expression (*via *insertional inactivation) results in a disruption of glucose metabolism and excretion of acetate. The authors proposed that UspA may have a direct effect on alteration of the flux of carbon through the primary metabolic pathways during arrested growth [57]. UspA has autophosphoryating activity [58] but is also phosphorylated by other proteins, including TypA [59]. If the parallels to other species hold, USPs in *M. tuberculosis *might affect the regulation of primary carbon metabolism, cause changes in the expression of a significant number of proteins, and generally modulate the metabolic shift to stasis. All eight of the Usp members found in *M. tuberculosis *show the ATP binding signature found in MJ0577 [60], for which there is a crystal structure with ATP bound [61]. Since ATP-competitive inhibitors are well-known, the USPs are very attractive targets. There is also a crystal structure of Rv1636 (unfortunately the lowest expressing member) opening up rational design approaches.

### *mprA*, *mprB *two component sensor

A second two component sensor kinase/transcriptional regulator, mprA/mprB, also appears to be involved in persistent infections. Biochemical characterization of the phosphorylated intermediates has established this as a *bona fide *two-component system [62]. Knockout experiments show a survival disadvantage in long term infection in mouse models, though the effect is tissue specific. Mice co-infected with wild type H37Rv *M. tuberculosis *and an mprA::Km^r ^mutant were analyzed after 147 days. In the spleen, 2% of the culturable *M. tuberculosis *were mutants, in the lung, 25% were mutants, and in the liver, 75% were mutants [63].

Bacterial sigma factors play a role in transcriptional initiation. A recent review summarizes the current state of knowledge of the *Mycobacterium *family [64]. Several sigma factors are up-regulated (*sigB, sigE, sigH, and sigF*) and there is a tantalizing functional connection between the *Bacillus subtilus *sigma factor *sigF *involved in sporulation and the *M. tuberculosis *homolog [65,66]. Cappelli *et al*. show *sigG *upregulation after 7 days in human macrophages, suggesting a role in adjusting transcription to survival inside the macrophage [67]. However, any novel anti-TB strategy based on the targeting of transcriptional components such as sigma-factors, would need to demonstrate some advantage over current rifampin therapy which also disrupts transcription through the inhibition of RNA polymerase activity. The *mprA*/*mprB *two-component system has been shown to directly regulate *sigB *and *sigE *[68]. The effect of the *mprA*::Km^r ^mutant was not as dramatic as could be hoped for, but if the link with the sigma factors and the metabolic shutdown in sporulation is substantiated, then the result of inhibition of *mprA *or *mprB *may be to reactivate *M. tuberculosis*.

### *relA *regulator/phosphorylase

Under conditions where amino acids or carbon are limiting, bacteria shift their metabolism via a series of well-studied pathways known as the stringent response. Disruption of the stringent response is a potential mechanism to force *M. tuberculosis *to exit from dormancy phase. In *M. tuberculosis*, the stringent response is mediated by *relA *(aka *Rel*_*Mtb*_, Rv2583c) which synthesizes and degrades hyperphosphorylated guanine nucleotides that in turn affect RNA polymerase and gene expression [69-72]. Dahl *et al*. compared *relA *knockouts with wild type under nutrient sufficient and starvation conditions and analyzed the expression patterns [69]. They concluded that *relA *globally down-regulates the cellular translation apparatus, including 54 of 58 ribosomal proteins. Fig. 4B shows the comparison of the expression profile of the down-regulated genes for the whole genome and the genes in Dahl *et al*. shown to be differentially regulated during starvation in *M. tuberculosis *wildtype but not in *relA *deletion mutants. *relA *showed a modest change in up-regulated expression, scoring 3.8, not far from the genome median of 3.1, and the positive scores do in fact come from the starvation conditions (Additional file 4). Hasan *et al*. [15] similarly scored *relA *low as a persistence target. However, despite the low score we feel that *relA *should be a good therapeutic target, especially if it disrupts the regulation of protein synthesis. It seems probable that *devR *and *relA *are working in concert, each responding to different environmental cues, to achieve the metabolic downshift to the non-replicative state.

### C_2 _metabolism

The dual stresses of an unbalanced nutrient pool and hypoxia place significant demands on the regulation of metabolites through the two-carbon pathways. Transcriptional analyses [29,73] suggest *M. tuberculosis *survives in the host on a nutrient pool rich in fatty acids and poor in carbohydrates [17,74]. Fatty acids are broken down to acetyl-CoA by the five-step beta-oxidation pathway. *M. tuberculosis *has 34 acyl-CoA synthases, 34 acyl-CoA dehydrogenases, 21 enoyl-CoA hydratases, six 3-hydroxyacyl-CoA dehydrogenases, and six acetyl-CoA C-acetyltransferases. There are over 250 lipid metabolizing enzymes in *M. tuberculosis *compared to 50 in *E. coli*, which underlines the importance of fatty acids as a nutrient source for this bacillus [17]. The postulate has been put forward that the main storage molecule for fatty acids are triacyl glycerols and 15 *M. tuberculosis *genes showed triacylglycerol synthase activity [75]. A knockout of the most active and highly upregulated synthase, Rv3130c, named *tgs1*, completely eliminates accumulation of triacyl glycerols in hypoxic *M. tuberculosis*. As the authors suggest, inhibition of *tgs1 *could lead to starvation of non-replicative cells, but may be compromised by the redundant activity of the other synthases.

### Pantothenate biosynthesis

Acetyl-coenzyme A (Ac-CoA) is a central intermediate in primary metabolism with roles in the TCA cycle as well as fatty acid and amino acid biosynthesis. The flux of carbon through Ac-CoA is particularly critical to non-replicating *M. tuberculosis*. Fatty acids, via breakdown to Ac-CoA and use of the glyoxylate shunt, provide the carbon for carbohydrate synthesis and acetyl-coenzyme A appears to be the gate through which much of the utilized carbon pool must pass. The *M. tuberculosis *membrane, being rich in unusual carbohydrates [76,77], is likely critically dependent upon this carbon flux through Ac-CoA for the maintenance of its integrity.

Targeting coenzyme A biosynthesis should disrupt the flux through several pathways. Supporting this notion are inhibitors of pantothenate kinase that suppress the growth of the low GC Gram-positive bacterium, *Staphylococcus aureus*, in a typical MIC assay [78]. However pantothenate biosynthesis knockouts in *M. tuberculosis *in mouse infection experiments suggest the operation of some salvage mechanism [79]. *PanC *and *panD *are two of the five genes needed for *de novo *biosynthesis. A double deletion mutant was constructed and tested in immunocompromised SCID and normal BALB/C mice. In both strains the *panCD *knockouts show greatly attenuated virulence. The SCID mice injected with the double mutant live seven times longer than those injected with the wild type H37Rv *M. tuberculosis*. The double mutant does not kill any of the BALB/c mice, even after 1 year. Yet in both cases there is significant bacterial load. The lungs of the SCID mice eventually reach >10^8 ^cfu, likely the cause of death. The lungs of the BALB/C mice reach almost 10^6 ^cfu after 2 weeks and show severe, diffuse granulamatous pneumonia; the infection is slowly cleared over the next months. The spleen and liver show similar pathologies. The mechanism that allows this persistence is not clear. The *panCD *knockouts are able to grow in SCID and BALB/C mice yet have greatly attenuated virulence. The authors suggest an unidentified permease is salvaging enough pantothenate to survive but not enough to cause disease. In order for a pantothenate biosynthesis inhibitor to be effective, the supply of exogenous pantothenate must be exhausted. This is possible since, as mentioned above, the granulomas formed in the mouse do not show the central caseating necrosis found in the human lung. Testing the *panCD *knockout in a model that closely replicates the human granuloma is critical to the validation of this pathway as a potential drug target.

### Redox balance

Under hypoxic conditions there is a lack of terminal electron acceptors and *M. tuberculosis *must reoxidize reduced metabolites to maintain viability. Maintaining redox balance, especially in the face of heavy use of fatty acids, is critically dependent upon the fate of the acetyl unit as it is either oxidized for energy, utilized for biosynthesis, or shunted off to intermediates to hold reducing equivalents. Eventually some of these reduced compounds must make their way to terminal electron acceptors.

Knockouts of the genes encoding two isocitrate lyase isoforms (*icl1 *and *icl2*) have shown that disruption of reactions close to or within these primary metabolic steps are effective and it has long been noted as a promising therapeutic target [74,80]. Its effectiveness in knockout experiments and with a prototype inhibitor (3-nitropropionate) is likely due to its role in enabling the utilization of the Ac-CoA pool for carbohydrate synthesis [81,74,80,82]. The 3-dimensional structure of the enzyme will also aid the discovery of inhibitors of ICL [83].

Genes for PEP (phosphoenolpyruvate) carboxykinase and a citrate synthase, both involved in carbohydrate metabolism, are also up-regulated. Three genes are annotated as citrate synthases, converting oxaloacetate and Ac-CoA to citrate and CoA. *Cit3 *shows significant up-regulation and could be an attractive target if the other two enzymes do not provide a functional alternative. Malate dehydrogenase (*mez*), another enzyme intimately involved in primary metabolism, shows modest up-regulation.

Alanine dehydrogenase has been suggested to be important both for the production of alanine (utilized in peptidoglycan) and for maintenance of the NAD pool [84,85]. The kinetic behavior of the enzyme strongly suggests that its primary function is to catalyze the formation of alanine. The *M. smegmatis *alanine dehydrogenase is also suggested to have glycine dehydrogenase activity [86]. If this holds for *M. tuberculosis*, inhibition of this enzyme would also disrupt the glyoxylate pool. The potential effect on a peptidoglycan precursor, redox balance, and carbohydrate synthesis coupled with its upregulation make this enzyme a viable target. The protein was also identified in a proteomics analysis of the Wayne model [87].

Another strategy to disrupt block redox balance is to attack the key carrier of reducing equivalents: NAD(P)^+^. Taking into account both avoidance of enzymes with close human orthologues and inclusion of those genes with high expression scores, the best target appears to be *nadR*. NadR is a bifunctional enzyme with nicotinamide-nucleotide adenylytransferase and ribosylnicotinamide kinase (RNK) activities.

### Respiratory chain

Another drug targeting opportunity created by hypoxia is the need for *M. tuberculosis *to utilize alternate terminal electron acceptors as the final step of respiration. Wayne and Sohasky [2] have proposed that nitrate is reduced by a nitroreductase (*narGHJ*) and is then excreted by a nitrite extrusion protein (*narK1, narK2, narK3*). This scheme is supported by the transcript levels of proteins in the respiratory chain. [27] Knockouts of *narG *in a SCID mouse model with *M. tuberculosis *BCG show a decreased virulence of the mutant compared to wild type [88], leaving open the possibility that blocking nitrate reduction could be effective against a truly hypoxic adapted *M. tuberculosis*. Nitrate reduction is controlled by the strongly up-regulated *narK2*, also a member of the *devR *regulon [89]. Sohaskey has shown that the presence of *narK2 *is responsible for the 75 fold difference in nitrite synthesis between actively growing, oxygenated cultures and hypoxic, non-replicative bacilli. This represents another strategy to take advantage of hypoxia.

Knockouts of the 'fused reductase' *narX *have no effect so this gene appears to be inactive [90]. Additionally, three genes which are significantly up-regulated contain the nitroreductase ortholgous group, COG0778 (Rv2032 (*acg*), Rv3131 and Rv3127). However the lack of nitrate reductase activity after a *narG *knockout argues against these as respiratory inhibition targets [90].

Moving up the reaction path leads to the proteins in the respiratory chain. There are several redox proteins that have high to moderate up-regulation scores and are likely involved as alternate electron carriers in the hypoxic environment: fumarate reductase (*frdA*), probable NAD(P)H dehydrogenases (Rv3054c, Rv0082, Rv1812c, and Rv1854c), and ferridoxin (*fdxA*). Ferridoxin is the most significantly up-regulated of the low-redox potential electron carriers [9]. Recent studies show that phenothiazines inhibit NADH:menaquinone oxidoreductase (Rv1854c, Rv0392c) and stop growth in acute models [91,92]. This class of inhibitor may force the respiratory chain into an oxidized state and *M. tuberculosis *to behave as if it were in an aerobic environment. The potential of this mechanism to signal the bacilli to switch its metabolism back to the replication, hence more vulnerable to existing antibiotics, is an intriguing therapeutic approach.

The liability with the respiratory chain targets is the potential for redundancy. It is unknown which or how many enzymes to inhibit for a lethal blockade. However, as mentioned above, inhibition may not be the best approach. Uncoupling electron transfer with generation of the proton motive gradient or phosphorylation of ATP should be rapidly lethal. Rv1812, Rv1854c and the fumarate reductase complex are believed to be components of the oxidative phosphorylation chain and could take advantage of the uncoupling strategy. The human orthologue to fumarate reductase, NP_004159, shows 35% identity and 51% similarity to the *M. tuberculosis *enzyme at the amino acid level, so there is the potential for toxicity that would have to be addressed.

### Downregulation of ATP synthesis

Since *M. tuberculosis *appears to have limited available ATP in dormancy phase, targeting ATP synthesis also looks to be a promising strategy. The newly discovered diarylyquinoline inhibitor of the ATP synthase proton pump [93] could be a valuable tool for establishing whether this approach would be effective against non-replicating cells. Compounds with similar modes of action as 2,4-dinirophenol that uncouple ATP synthesis from proton movement may also work provided they could be made selective for *M. tuberculosis *[94,95]. It is unclear whether targeting processes that utilize ATP would result in cell death. It is possible that they are occurring so slowly already (due to low availability of ATP) that inhibition would have a minor effect. An alternative approach would be to deplete available ATP stores. A compound that caused a futile ATP cycle could be very effective in killing dormant cells. For example if an ATP dependent transporter could be reduced so as to be only 1% efficient, this could drain the ATP pool and leave the bacterium unable to recover.

### Sulfate transport and metabolism

Wooff *et al*. [96] have studied sulfate transport knockouts in *M. bovis *in detail. The sulfate transporter is made up of the proteins from *cysA1*, *cysT*, *cysW*, and *subI*. Transposon insertions into *cysA1 *or *subI *yield methionine auxotrophs completely unable to import sulfate which indicates that *cysA1 *is the sole sulfate transporter. They conclude that *cysA2, and cysA3 *are in fact thiosulfate sulfurtransferases. *M. tuberculosis *has two additional genes that are up-regulated and also annotated as possible sulfate transporters (Rv1707 and Rv1739c); it is not known whether these are active.

Wooff *et al*. also inoculated BALB/c mice with the *cysA1 *auxotroph and found no difference in growth compared to wild type, suggesting that the mutant was able to survive well using exogenous sources of methionine. Recently, it has shown that *M. tuberculosis *can convert methionine into cysteine [97]. The expression results analyzed here imply that sulfur is deficient within *M. tuberculosis*. Whether this situation can be remedied by scavenging methionine is likely dependent upon the nutrient pool available in the local environment of the granuloma.

Once sulfate is transported in the cell, it is activated by conversion into adenosine 5'-phosphosulfate (APS) and 3'-phosphoadenosine 5'-phosphosulfate (PAPS). The genes for these enzymes (*cysD *and *cysN*) are significantly up-regulated. *M. tuberculosis CysN*, also known as *cysNC*, contains two fused activities, sulfate adenylyltransferase (*cysN*) and adenylylsulfate kinase (*cysC*) [98]. These genes have been cloned and the recombinant enzymes are functional and produce PAPS from sulfate, ATP and GTP [98,99]. Recent work has shown that in *M. tuberculosis *APS, not PAPS, is utilized for cysteine and methionine biosynthesis [100]. Several genes in this pathway are up-regulated: *cysD*, *cysN *(transferase), *metC*, and *cysK2*. The same issue arises here as with the transporters: will methionine salvage outflank the inhibitory blockade? As above this remains to be tested with a necrotic granuloma.

There is an additional possibility, however. Another role of sulfate is in the synthesis of sulfated lipids. (See reference [101] for a review of sulfate metabolism in *M. tuberculosis*.) Sulfated lipids are synthesized from PAPS and known targets to date are *cysC *(kinase domain) and Rv1373 [100]. Inhibition of sulfolipid synthesis may be effective in the early phases of infection [102]; it is not yet known what the role of sulfated lipids is in persistent infection, but the hope is that the up-regulation of the genes for APS, PAPS and sulfolipid synthesis are indicative of a critical function.

### Other transporters

*CtpF *and *ctpG *are annotated as probable ATP dependent cation transporters. If the annotations are in fact correct then these are promising targets. They have the advantage of avoiding the salvage problem above and opening up the opportunity for futile hydrolysis of ATP. However, the high similarity of these two proteins with human orthologs could provide challenges in the development of *M. tuberculosis *selective inhibitors (Additional file 4).

### Downregulation of protein synthesis

Rifampin, a transcriptional inhibitor, is one of the few drugs effective against *M. tuberculosis *in the NRP-2 phase of the Wayne model [103]. Rifampin thus serves as an important benchmark: it has efficacy against persistent *M. tuberculosis*, so any strategy that does not have a rationale for a shorter therapy should not make the list of new targets. We put proteins synthesis inhibitors into this category. This is borne out in clinical trials: streptomycin, an inhibitor of protein synthesis the binds the 30S ribosome, is not as effective as rifampin throughout a long treatment period [104,35].

### Ribonucleotide reductase

The effectiveness of inhibiting ribonucleotide reductase in actively growing cells is shown by Dawes *et al*. [105]. The large and small subunits of the type Ib reductase is encoded by *nrdE *and *nrdF2*, respectively. Knockout of *nrdF2 *is lethal, both validating this gene as a target, and showing that neither *nrdF1 *nor *nrdZ *could substitute. In contrast to our analysis (which show up-regulation of all the *nrd *genes), quantitative RT-PCR studies of *nrdEF2 *in the Wayne model shows a 10 fold decrease in expression from log phase to NRP-1, followed by a 2–3 fold increase from there as the cells progress into NRP-2. Dawes *et al*. also found surprising results with knockouts of *nrdZ *which encodes a putative class II reductase that is also up-regulated by *devR*. The expected scenario where *nrdEF2 *is down-regulated and *nrdZ *takes over during hypoxia was not observed. *NrdZ *knockouts had no effect on survival in the Wayne model or in B6D2/F_1 _mice, even after 14 months. So it appears that *nrdEF2 *is still supplying the needed pool of deoxyribonucleotides.

With regard to the main focus of our analysis, the real question is one of rate: is the rate of DNA damage to non-replicating *M. tuberculosis *high enough so that blocking repair will shorten therapy? The main impetus for developing ribonucleotide reductase inhibitors will certainly be to kill actively dividing cells. They may have the additional benefit of preventing cells from reactivating or leaving cells with a damaged genome to die.

### Isoprene biosynthesis

As shown in Fig. 5, *M. tuberculosis *solely utilizes the pyruvate/glyceraldehyde phosphate pathway for isopentenyl-pyrophosphate biosynthesis and lacks the mevalonate pathway found in eukaryotes and some low GC Gram positive organisms [76]. Isopentenyl-pyrophosphate is a precursor in the synthesis of decaprenyl phosphate. Decaprenyl phosphate is a 50-carbon lipid that has at least 2 functions in the productions of the mycolic acid-arabinogalactan-peptidoglycan complex of the cell wall [76]. Nearly all of the enzymes of the cell wall biosynthetic pathway show low up-regulation scores (typically 0 – 4; as an example, *murA, murB, murC, murD, murE, murF*, and *murG *score 1.5, 0, 0, 2.2, 3.9, 0.3, and 3.8 respectively). Cell wall biosynthesis is already targeted by isoniazid (which is most effective in the first few days of TB treatment) [104], and the expression scores are low, so the argument can be made that targeting the cell wall is not interesting in the context of attacking non-replicating *M. tuberculosis*. However a recent review argues that maintenance of the membrane in persistent *M. tuberculosis *is important for long term viability, but the biochemical mechanisms are very difficult to determine [106]. The function of decaprenyl phosphate as a carrier molecule may be more disruptive to downstream processes. We believe that this coupled with the knockout and up-regulation seen in the long term experiments makes this pathway a viable target.

### Other Targets

#### lysine-ε-aminotransferase

Lysine is biosynthesized starting with aspartate and pyruvate, the so-called diaminopimilate pathway. The data here shows that this pathway is significantly downregulated. An altenative pathway exists in fungi, which starts with Ac-CoA and 2-keto-glutarate. The final enzyme in the fungal pathway, lysine-ε-aminotransferase, has been identified in *M. tuberculosis *while the other nine enzymes are either absent or yet to be annotated. Lysine-ε-aminotransferase is also found in several bacterial and fungal species. Its role in non-replicative metabolism is not clear. The high up-regulation score makes this an intriguing potential target. A crystal structure of this enzyme has recently been reported which could facilitate rational drug design [107].

#### Membrane protease regulating envelope composition

Rv2869c has been identified as an intramembrane cleaving protease (iCLIP) that regulates the composition of mycolic acids and some phosphatidylinositol mannosides in the *M. tuberculosis *cell envelope, as well as having transcriptional effects on other lipid metabolizing enzymes [108]. A deletion of the protease had no effect on cell counts in infected mice after 24 h indicating that it is not critical for the acute phase of infection, however after 22 weeks the bacterial load in the lung was down 10,000 fold compared to wild type. The up-regulation score for this enzyme is not even at the genomic median, but the knockout results, as well as the lack of a closely related human homolog, make a compelling case for this as a therapeutic target.

### Chaperonins/HSP

Wayne and Sohaskey have suggested that the decreased effectiveness of rifampin in the non-replicative state in the Wayne model could be due in part to the stabilizing effect of the chaperonins [2]. Thus a combination therapy of rifampin and a chaperonin inhibitor has the potential to shorten the therapeutic regimen. Alpha-crystallin 1 (Rv031c, *hspX*, *acr1*) has a large molecular chaperone domain [24] and is among the genes most highly induced in the *devR *regulon. The biology of alpha-crystallin 1 is complex as evidenced by the effect on growth when the genes are knocked out. Recent work has shown more rapid initial growth and increased colony counts in mouse lung and spleen in *acr1 *knockouts [109,110]. These findings suggest that *acr1 *has a role in the growth rate both during the initial stage of infection and long term persistence. The role of *acr1 *as a growth modulator raises the attractive possibility that interfering with its function could cause a beneficial switch to active replication.

The second of two alpha-crystallins in *M. tuberculosis*, encoded by the gene *acr2*, has one of the highest expression scores, consistent with Stewart *et al*. [111] where it was up-regulated at every stage of infection. A knockout of the *acr2 *gene had no significant effect on bacterial load in the mouse lung or spleen, though the mice did not experience as significant weight loss as those infected with wild type. An interesting experiment would be the effect of dual knockouts of both *acr1 *and *acr2 *genes in the presence of rifampin.

*M. tuberculosis *has other chaperonins; *groEL1 *and *groEL2 *show moderate up-regulation scores and should also work in concert with rifampin. A concern with these two proteins is that there are reasonably close mammalian orthologues. A human chaperonin (NP_955472) has 41% identity and 65% similarity with *groEL1*, and *groEL2 *(for which there is a crystal structure [112]) is 46% identical and 68% similar to another human chaperonin (NP_002147). Again there is the problem of redudancy, with at least four proteins capable of stabilizing proteins during persistence.

## Conclusion

Here we integrated a number of data sets that utilized DNA microarrays covering spectrum of experimental conditions into a single analysis of dormant phase *M. tuberculosis*. Drug targets to specifically attack non-replicative bacilli emerging from our analysis were several regulatory elements that have the potential to force *M. tuberculosis *back into replicative mode. Other targets take advantage of the presumed hypoxic environment to state to disrupt redox balance, an altered respiratory chain, or the likely scarcity of ATP.

A recent paper was able to examine the transcriptional profile of *M. tuberculosis *from human lung samples [113]. The patients had active disease and were on chemotherapy so some of the granulomas were breaking up as part of reactivation. As a result a fraction of the cells were exposed to oxygen *in vivo *and possibly post excise as the resected samples were exposed to air. This could explain the up-regulation of a number of genes involved in aerobic metabolism. While a very important study we believe that these samples may be quite heterogeneous and might not entirely reflect the dormant phase.

The advent of large scale genomics covering not only the genomes of multiple bacterial species but also various strains within a pathogenic species, has lead to the application of comparative genomics analysis to derive lists of high priority gene targets. Genes can be ranked by a number of different criteria such as divergence from human genes; essentiality for both *in vitro *and infectious viability; homology across known pathogens; importance to pathogenic life cycle; tractability to small molecule inhibitors and so on. In reality, some seemingly obvious criteria have little pharmacological relevance. For example, the antibiotic rifampicin targets DNA-directed RNA polymerase which is highly conserved across all species including prokaryotes and humans yet it is a key member of the anti-TB compound quartet. While bioinformatics derived lists of prioritized genes can provide important starting points for new target discovery and to help organize genomic data [15], decisions on which TB drug development strategies to pursue requires additional context about pathogen biology, biochemistry, pharmacology and pathogenicity. Using this approach, we have suggested several potential drug development strategies against dormant phase *M. tuberculosis *which target key biosynthetic pathways.

## Methods

The data sets and scoring weights compiled for this analysis are listed in Table 1. A zero to five scoring system was developed that utilized both the relevance of the experimental conditions to the dormant state and the rank order of expression.

The maximum score for a particular experimental dataset was adjusted based on our judgement of relevance to the clinical occurrence of dormancy phase *M. tuberculosis *infections. For studies with multiple time series sampling, increasing weight was given to later time-points samples. For example Wayne and Hayes [8] have shown two phases of metabolic shiftdown: NRP-1 (characterized by tolerance to metronidazole, greatly slowed growth, and thickening of cell walls) and NRP-2 (characterized by sensitivity to metronidazole and absence of growth) [114]. In order to take into account both phases, but weigh the scoring toward NRP-2, which is likely more relevant to clinical dormancy, genes in NRP-2 received higher scores (e.g. 5 points maximum) than genes in NRP-1 (e.g. 2 or 3 points maximum).

Once the maximum score was set for a particular experiment, the genes were ordered from highest to lowest based on expression ratio (fold expression in the experimental condition versus cells in log-phase liquid culture). The highest scoring gene received the maximum score (listed in column 3 of Table 1. (e.g. 5, 4 ..., 1 point)). The score was decreased by 0.005 points for each gene in order until zero, or the end of the data set was reached. Thus when the maximum score was 4 points, the 100^th ^ranked gene would receive a score of 3.500. For a maximum score of 5 points, 1000 genes or 25% of the MTB genome received a score. For experiments where data from multiple time points were collected, the maximum score across all time points was used as the final score.

For example the up-regulation score of Rv3133c, *devR*, was calculated by summing the maximum scoring time-point (underlined) from the following studies (score:timepoint):

Hampshire *et al*., 2004 [30] (0:18 d, 0:49 d, 3.57:62 d, 0.185:76 d);

Muttucumaru *et al*., 2004 [115] (1.915:7 d, 3.915:14 d);

Voskuil *et al*. 2004 [31] (0:4 d, 1.875:6 d, 1.895:8 d, 2.860:10 d, 2.815:12 d, 3.820:14 d, 3.07:20 d, 0:30 d, 3.325:80 d);

Schnappinger *et al*. 2003 [29] (0:24 h (naïve, ampl.), 0:24 h (naïve, oligo.), 0:48 h (naïve, ampl.)), 4.870:24 h (activated, ampl.), 4.800:24 h (activated, oligo.), 4.185:48 h (activated, ampl.).

In the remaining 3 studies [116-118]), this gene scored zero. Therefore, the up-regulation score of Rv3133c, *devR *equaled 16.175.

The scores for down-regulated expression were collected in a similar fashion, except that genes were ordered from lowest to highest and scores were negative. For the transposon-based knockout experiments, genes which had no effect on growth received a score of zero.

## Competing interests

The author(s) declare that they have no competing interests.

## Authors' contributions

DJM performed the analysis and wrote the draft manuscript. JRB conceived the study and co-wrote the final manuscript.

## Pre-publication history

The pre-publication history for this paper can be accessed here:



## Supplementary Material

Additional file 1 Scoring and annotations for all predicted open reading frames in the *Mycobacterium tuberculosis *genome. Searchable table of scoring results for all protein coding genes in the *Mycobacterium tuberculosis *genome. Click here for file

Additional file 2 Scoring and annotations for Dev/Dos regulon genes. Searchable table of scoring results for all protein coding genes activated by the Dev/Dos regulon. Click here for file

Additional file 3 Scoring and annotations for ATP synthase subunit genes. Searchable table of scoring results for genes encoding various ATP subunits in the *Mycobacterium tuberculosis *genome. Click here for file

Additional file 4 Pathways and associated genes which are potential TB dormancy phase targets. Genes which are likely the best targets against dormancy phase of infectious *Mycobacterium tubercuolosis *categorized by pathways and annotated with known structures and human homologs, if available. Click here for file
