# Supplementary material for: Identification of gene targets against dormant phase Mycobacterium tuberculosis infections
Source: BMC Infect Dis. 2007 Jul 26;7:84. doi: 10.1186/1471-2334-7-84 (PMC1950094; doi:10.1186/1471-2334-7-84)
Supplement: Additional file 4 — Pathways and associated genes which are potential TB dormancy phase targets. Genes which are likely the best targets against dormancy phase of infectious Mycobacterium tubercuolosis categorized by pathways and annotated with known structures and human homologs, if available. [file 1471-2334-7-84-S4.doc]

Additional file 4. Pathways and associated genes which are potential TB dormancy phase targets.

| **Rv number*** |  | **Up-regula-tion Score** | **Down-regula-tion Score** | **Growth-attenua-tion Score** | **Gene ID** | **Protein ID** | **†Brief Annotation** |
| --- | --- | --- | --- | --- | --- | --- | --- |
| **devS/devT devR (dosR) Two component response regulator (dosR regulon)** | | | | | | | |
| Rv3132c |  | 13.815 | -4.935 | 7.495 | devS | NP_217648.1 | sensor histidine kinase |
| Rv3133c |  | 16.175 | -3.085 | 0 | devR | NP_217649.1 | two-component response regulator [=1ZLK, =1ZLJ] |
| Rv2027c |  | 3.47 | -3.99 | 0 | devT | NP_216543.1 | sensor histidine kinase |
| **mprAB 2 component response regulator** | | | | | |  |  |
| Rv0981 |  | 13.21 | -3.56 | 0 | mprA | NP_215496.1 | two-component response regulator [=1YS6] |
| Rv0982 |  | 8.14 | -0.485 | 3.48 | mprB | NP_215497.1 | sensor histidine kinase [1YS3, 2C2A] |
| **triacylglycerol biosynthesis** | | | |  |  |  |  |
| Rv3130c |  | 20.135 | 0 | 0 | tgs1 | NP_217646.1 | triacylglycerol synthase |
| Rv3734c |  | 3.635 | -6.835 | 0 | tgs2 | NP_218251.1 | triacylglycerol synthase |
| Rv3234c |  | 1.8 | -5.8 | 0 | tgs3 | NP_217751.1 | triacylglycerol synthase |
| Rv3088 |  | 7.325 | 0 | 0 | tgs4 | NP_217604.1 | triacylglycerol synthase |
| Rv1760 |  | 4.51 | 0 | 0 |  | NP_216276.1 | triacylglycerol synthase |
| Rv2285 |  | 0 | -0.77 | 0 |  | NP_216801.1 | triacylglycerol synthase |
| Rv0221 |  | 1.995 | -5.015 | 0 |  | NP_214735.1 | triacylglycerol synthase |
| Rv3740c |  | 2.69 | -1.675 | 0 |  | NP_218257.1 | triacylglycerol synthase |
| Rv3087 |  | 3.315 | -4.785 | 4.64 |  | NP_217603.1 | triacylglycerol synthase |
| Rv3371 |  | 9.255 | -2.515 | 4.945 |  | NP_217888.1 | triacylglycerol synthase |
| Rv3480c |  | 0 | -7.355 | 4.97 |  | NP_217997.1 | triacylglycerol synthase |
| Rv3233c |  | 0 | -8.12 | 0 |  | NP_217750.1 | triacylglycerol synthase |
| Rv1425 |  | 0 | -2.66 | 0 |  | NP_215941.1 | triacylglycerol synthase |
| Rv0895 |  | 4.015 | -2.575 | 0 |  | NP_215410.1 | triacylglycerol synthase |
| Rv2484c |  | 2.04 | -3.325 | 0 |  | NP_217000.1 | triacylglycerol synthase |
| **pantothenate biosynthesis** | | | |  |  |  |  |
| Rv1820 |  | 0.765 | -0.35 | 0 | ilvG | NP_216336.1 | acetolactate synthase II [NP_006835, 5e-72, 2C31] |
| Rv3003c |  | 3.185 | -3.495 | 4.75 | ilvB1 | YP_177917.1 | acetolactate synthase I large subunit [NP_036392, 2e-44, 1JSC] |
| Rv3470c |  | 8.27 | -2.7 | 0 | ilvB2 | NP_217987.1 | acetolactate synthase large subunit NP_006835, 4e-26, 1JSC] |
| Rv3002c |  | 7.19 | -6.545 | 4.275 | ilvN | NP_217518.1 | acetolactate synthase I small subunit [2FGD] |
| Rv3509c |  | 2.065 | -7.875 | 0 | ilvX | NP_218026.1 | probable acetohydroxyacid synthase I large subunit [1YNO] |
| Rv3001c |  | 3.045 | -6.77 | 3.7 | ilvC | NP_217517.1 | ketol-acid reductoisomerase [1NP3] |
| Rv0189c |  | 4.415 | 0 | 3.37 | ilvD | NP_214703.1 | dihydroxy-acid dehydratase [2GP4] |
| Rv2225 |  | 9.315 | -1.985 | 2.845 | panB | NP_216741.1 | 3-methyl-2-oxobutanoate hydroxymethyltransferase [1OY0] |
| Rv2210c |  | 0 | -4.715 | 3.63 | ilvE | NP_216726.1 | branched-chain-amino-acid transaminase [NP_005495, 5e-61, 2COG] |
| Rv2573 |  | 2.22 | -6.76 | 0 |  | NP_217089.1 | putative 2-dehydropantoate 2-reductase [2EW2] |
| Rv3602c |  | 1.555 | 0 | 3.815 | panC | NP_218119.1 | pantoate-[beta]-alanine ligase [=1MOP] |
| Rv3601c |  | 0 | -2.135 | 0 | panD | NP_218118.1 | aspartate 1-decarboxylase [2C45] |
| Rv1092c |  | 0 | -6.31 | 8.025 | coaA | NP_215608.1 | pantothenate kinase [2GES] |
| Rv1391 |  | 2.205 | -5.995 | 3.42 | dfp | NP_215907.1 | flavoprotein [NP_068595, 3e-15, 1U7U] |
| Rv2965c |  | 2.22 | -2.805 | 0 | kdtB | NP_217481.1 | lipopolysaccharide core biosynthesis protein [1TFU] |
| Rv1631 |  | 0 | -5.93 | 3.61 | coaE | NP_216147.1 | conserved hypothetical protein [NP_079095, 3e-22, 1VHL] |
| Rv2523c |  | 0 | -4.875 | 0 | acpS | NP_217039.1 | CoA:apo-[ACP] pantethienephosphotransferase [1F7T] |
| **Isoprene biosynthesis** | | | |  |  |  |  |
| Rv2682c |  | 0 | -0.23 | 3.335 | dxs1 | YP_177898.1 | 1-deoxy-D-xylulose 5-phosphate synthase [NP_115512, 3e-17, 2O1S] |
| Rv1086 |  | 9.25 | -0.6 | 0 |  | NP_215602.1 | conserved hypothetical protein [NP_079163, 6e-29, 1F75] |
| Rv3379c |  | 9.465 | -4.31 | 0 | dxs2 | NP_217896.1 | unknown transketolase, interrupted by IS6110 [NP_115512, 7e-16, 2O1X] |
| Rv2870c |  | 4.275 | 0 | 0 | dxr | NP_217386.2 | conserved hypothetical protein [=2C82] |
| Rv3582c |  | 11.555 | -1.47 | 4.565 | ispD | NP_218099.1 | conserved hypothetical protein [1VPA] |
| Rv1011 |  | 3.59 | -5.15 | 3.165 | ispE | NP_215527.1 | conserved hypothetical protein [1UEK] |
| Rv3581c |  | 10.78 | 0 | 3.795 | ispF | NP_218098.1 | conserved hypothetical protein [1H47] |
| Rv2868c |  | 5.075 | -4.515 | 0 | gcpE | NP_217384.1 | essential gene of unknown function |
| Rv1110 |  | 4.145 | -2.335 | 0 | lytB2 | YP_177788.1 | very similar to LytB |
| Rv3382c |  | 0.13 | 0 | 0 | lytB1 | YP_177967.1 | LytB protein homologue |
| **Universal stress proteins** | | | |  |  |  |  |
| Rv2005c |  | 15.5 | -1.28 | 0 |  | NP_216521.1 | conserved hypothetical protein |
| Rv2623 |  | 20.825 | -2.29 | 2.9 |  | NP_217139.1 | conserved hypothetical protein |
| Rv3134c |  | 19.29 | -2.46 | 0 |  | NP_217650.1 | conserved hypothetical protein |
| Rv2624c |  | 17.475 | -0.185 | 0 |  | NP_217140.1 | conserved hypothetical protein |
| Rv1996 |  | 13.63 | -2.71 | 0 |  | NP_216512.1 | conserved hypothetical protein |
| Rv2028c |  | 9.605 | -3.085 | 0 |  | NP_216544.1 | conserved hypothetical protein [1WJG] |
| Rv2026c |  | 7.19 | 0 | 2.485 |  | NP_216542.1 | conserved hypothetical protein [|NP_079368, 4e-27, 1H54] |
| Rv1636 |  | 0.505 | -7.255 | 0 |  | NP_216152.1 | conserved hypothetical protein [1TQ8] |
| **Sulfate fixation** | | |  |  |  |  |  |
| Rv1285 |  | 17.68 | 0 | 4.255 | cysD | NP_215801.1 | ATP:sulphurylase subunit 2 [NP_003866, 6e-21] |
| Rv1286 |  | 12.78 | 0 | 3.755 | cysN | NP_215802.1 | ATP:sulphurylase subunit 1 [NP_005434, 3e-45, 1ZUN] |
| Rv3340 |  | 9.995 | 0 | 0 | metC | NP_217857.1 | cystathionine [beta]-lyase [NP_001893, 3e-56, 2CTZ] |
| Rv0848 |  | 12.8 | 0 | 0 | cysK2 | YP_177762.1 | putative cysteine synthase [NP_000062, 6e-26, 1VE1] |
| Rv1373 |  | 4.07 | -2.935 | 0 |  | NP_215889.1 | slight similarity to sulfotransferases [NP_814444, 2e-12] |
| Rv2392 |  | 1.215 | -9.135 | 3.17 | cysH | NP_216908.1 | 3'-phosphoadenylylsulfate (PAPS) reductase [2GOY] |
| **Sulfate transporter** | | |  |  |  |  |  |
| Rv1707 |  | 11.61 | 0 | 0 |  | NP_216223.1 | probable sulphate permease [NP_075062, 5e-25 ] |
| Rv1739c |  | 10.5 | -5.47 | 0 |  | NP_216255.1 | possible sufate transporter [NP_075062, 8e-57 ] |
| Rv2398c |  | 7.73 | -2.995 | 3.615 | cysW | NP_216914.1 | sulphate transport system permease protein [2ONK] |
| Rv2399c |  | 9.59 | -0.735 | 3.27 | cysT | NP_216915.1 | sulphate transport system permease protein [2ONK] |
| **NAD biosynthesis** | | |  |  |  |  |  |
| Rv0212c |  | 11.785 | 0 | 0 | nadR | NP_214726.1 | similar to E.coli NadR [NP_079509, 6e-07, 1LW7] |
| **Nitroreductase** | | |  |  |  |  |  |
| Rv2032 |  | 19.4 | -2.755 | 0 | acg | NP_216548.1 | conserved hypothetical protein |
| Rv3131 |  | 16.055 | -3.31 | 3.345 |  | NP_217647.1 | conserved hypothetical protein |
| Rv3127 |  | 14.055 | -6.615 | 0 |  | NP_217643.1 | conserved hypothetical protein |
| Rv1736c |  | 6.795 | -3.145 | 0 | narX | NP_216252.1 | fused nitrate reductase [1Q16] |
| **Respiratory chain** | | |  |  |  |  |  |
| Rv3054c |  | 15.8 | -0.85 | 0 |  | NP_217570.1 | conserved hypothetical protein [NP_000895, 2e-20, 1RTT] |
| Rv0082 |  | 11.51 | -2.55 | 0 |  | NP_214596.1 | probable oxidoreductase subunit [NP_077718, 1e-19, 2FUG] |
| Rv1812c |  | 7.565 | -1.88 | 0 |  | NP_216328.1 | probable dehydrogenase |
| Rv1854c |  | 7.39 | -3.525 | 0 | ndh | NP_216370.1 | probable NADH dehydrogenase [NP_116186, 5e-10, 1XHC] |
| Rv1552 |  | 12.925 | 0 | 0 | frdA | NP_216068.1 | fumarate reductase flavoprotein subunit [NP_004159, 4e-95, 1KF6] |
| Rv1553 |  | 5.96 | -2.81 | 0 | frdB | NP_216069.1 | fumarate reductase iron sulphur protein [1KF6] |
| Rv1554 |  | 6.32 | -2.675 | 0 | frdC | NP_216070.1 | fumarate reductase 15kD anchor protein |
| Rv1555 |  | 4.735 | -2.72 | 0 | frdD | NP_216071.1 | fumarate reductase 13kD anchor protein |
| **nitrite extrusion protein** | | | |  |  |  |  |
| Rv1737c |  | 13.04 | -2.915 | 0 | narK2 | NP_216253.1 | nitrite extrusion protein |
| **protease regulating envelope composition** | | | | | |  |  |
| Rv2869c |  | 2.22 | -1.76 | 2.585 |  | NP_217385.1 | probable integral membrane protein [1KY9] |
| **cation transport ATPase** | | | |  |  |  |  |
| Rv1997 |  | 16.165 | -3.32 | 0 | ctpF | NP_216513.1 | probable cation transport ATPase [NP_001001486, 1e-150, 2EAR] |
| Rv1992c |  | 14.45 | 0 | 0 | ctpG | NP_216508.1 | probable cation transport ATPase [NP_000044, 1e-70, 2B8E] |
| **Chaperonins/hsp** | | |  |  |  |  |  |
| Rv0251c |  | 21.935 | -1.81 | 0 | hsp | NP_214765.1 | possible heat shock protein [1GME] |
| Rv2031c |  | 15.78 | 0 | 0 | hspX | NP_216547.1 | 14kD antigen, heat shock protein Hsp20 family [1GME] |
| Rv0353 |  | 6.265 | 0 | 0 | hspR | NP_214867.1 | heat shock regulator |
| Rv0563 |  | 15.86 | -2.125 | 0 | htpX | NP_215077.1 | probable (transmembrane) heat shock protein [NP_005848, 6e-26, 1P7B] |
| Rv0384c |  | 15.625 | -0.37 | 4.025 | clpB | NP_214898.1 | heat shock protein [NP_004784, 1e-39, 1JBK] |
| Rv0440 |  | 8.565 | -9.995 | 3.655 | groEL2 | NP_214954.1 | 60 kD chaperonin 2 [NP_955472, 1e-138, =1SJP] |
| Rv3417c |  | 8.74 | -3.985 | 2.31 | groEL1 | NP_217934.1 | 60 kD chaperonin 1 [NP_955472, 1e-117, =1SJP] |
| Rv3418c |  | 6.92 | -6.04 | 3.895 | groES | NP_217935.1 | 10 kD chaperone [NP_002148, 3e-12, =1HX5] |
| Rv3610c |  | 7.115 | -2.995 | 3.23 | ftsH | NP_218127.1 | inner membrane protein, chaperone [NP_006787, e-120, 2CE7] |
| **Ribonucleotide reductase** | | | |  |  |  |  |
| Rv0233 |  | 9.05 | 0 | 0 | nrdB | NP_214747.1 | ribonucleoside-diphosphate reductase B2 [1XSM] |
| Rv3051c |  | 7.085 | -5.02 | 4.415 | nrdE | NP_217567.1 | ribonucleoside diphosphate reductase [alpha] chain [NP_001024, 8e-46, 1PEM, 4R1R] |
| Rv1981c |  | 8.98 | -1.695 | 0 | nrdF1 | YP_177853.1 | ribonucleotide reductase small subunit [1KGN] |
| Rv3048c |  | 15.845 | -0.49 | 2.085 | nrdF2 | YP_177921.1 | ribonucleoside-diphosphate small subunit [=1UZR] |
| Rv3053c |  | 9.72 | -3.08 | 0 | nrdH | NP_217569.1 | glutaredoxin electron transport component of NrdEF [1R7H] |
| Rv3052c |  | 10.97 | -3.145 | 0 | nrdI | NP_217568.1 | NrdI/YgaO/YmaA family [1RLJ] |
| **lysine-e-aminotransferase** | | | |  |  |  |  |
| Rv3290c |  | 19.385 | 0 | 0 | lat | NP_217807.1 | lysine-[epsilon] aminotransferase NP_000654, 1e-39, =2CIN] |
|  | Redox balance | | |  |  |  |  |
| Rv0467 |  | 12.25 | -1.385 | 0 | icl | YP_177728.1 | isocitrate lyase [=1F61] |
| Rv1915 |  | 5.545 | -6.31 | 0 | aceAa | NP_216431.1 | isocitrate lyase, [alpha] module [1F61, 1F8IA] |
| Rv1916 |  | 2.605 | -4.555 | 0 | aceAb | NP_216432.1 | isocitrate lyase, [beta] module |
| Rv2780 |  | 15.115 | 0 | 0 | ald | NP_217296.1 | L-alanine dehydrogenase [NP_036475, 3e-32, 1PJB, 1F8G] |
| Rv0211 |  | 8.635 | -1.875 | 0 | pckA | NP_214725.1 | phosphoenolpyruvate carboxykinase [NP_002582, e-176, 1KHB] |
| Rv1131 |  | 11.92 | -1.675 | 0 | gltA1 | NP_215647.1 | citrate synthase 3 [NP_004068, 5e-14, 1A59] |
| Rv2332 |  | 8.69 | 0 | 0 | mez | NP_216848.2 | probable malate oxidoreductase [NP_002386, 3e-96, 1LLQ] |
| **sigma factors** | | |  |  |  |  |  |
| Rv0182c |  | 4.1 | -4.59 | 0 | sigG | NP_214696.1 | sigma-70 factors ECF subfamily [1H3L, 1OR7] |
| Rv0445c |  | 2.335 | -12.13 | 0 | sigK | NP_214959.1 | ECF-type sigma factor |
| Rv0735 |  | 0.805 | -4.64 | 0 | sigL | NP_215249.1 | sigma-70 factors ECF subfamily |
| Rv1189 |  | 2.2 | -2.085 | 0 | sigI | NP_215705.1 | ECF family sigma factor |
| Rv1221 |  | 13.005 | -3.67 | 0 | sigE | NP_215737.1 | ECF subfamily sigma subunit [1OR7] |
| Rv2069 |  | 6.48 | -5.88 | 0 | sigC | NP_216585.1 | ECF subfamily sigma subunit [2O7G] |
| Rv2703 |  | 0.47 | -3.82 | 3.33 | sigA | NP_217219.1 | RNA polymerase sigma factor (aka MysA, RpoV) [NP_005372|NP_005372, 3e-13, 1L9Z] |
| Rv2710 |  | 18.915 | 0 | 2.005 | sigB | NP_217226.1 | RNA polymerase sigma factor (aka MysB) [1IW7] |
| Rv3223c |  | 9.98 | -1.76 | 4.885 | sigH | NP_217739.1 | ECF subfamily sigma subunit [1H3L] |
| Rv3286c |  | 6.365 | -2.07 | 0 | sigF | NP_217803.1 | ECF subfamily sigma subunit [1L0O] |
| Rv3328c |  | 0 | -1.86 | 0 | sigJ | NP_217845.1 | similar to SigI, ECF family [1OHS] |
| Rv3414c |  | 1.66 | -4.91 | 0 | sigD | NP_217931.1 | ECF subfamily sigma subunit [1OR7] |
| Rv3911 |  | 4.265 | -4.19 | 0 | sigM | NP_218428.1 | probable sigma factor, similar to SigE [1OR7] |
| **relA** |  |  |  |  |  |  |  |
| Rv2583c |  | 3.775 | -3.995 | 0 | relA | NP_217099.1 | (p)ppGpp synthase I [NP_940929, 5e-13, 1VJ7] |

*Multiple genes in the same pathway are included for score comparisons. †In brackets are the closest human ortholog, BLASTP [123] expect (E) value, and relevant 3-D structure (pdb entry), if any. An E-value cutoff of 1e-10 was used for inclusion of a human ortholog. An equal sign before the pdb entry indicates the protein is from *M. tuberculosis.*
